# Adaptive Strategies to Biotic Stress in Qatar’s Native Flora

**DOI:** 10.3390/life15111645

**Published:** 2025-10-22

**Authors:** Bassam Taha Yasseen, Roda Fahad Al-Thani

**Affiliations:** Department of Biological and Environmental Sciences, College of Arts and Sciences, Qatar University, Doha P.O. Box 2713, Qatar; ralthani@qu.edu.qa

**Keywords:** abiotic factors, adaptation, avoidance, biotic stress, chemical constituents, morphology, physical barriers, resistance

## Abstract

Qatar’s arid and semi-arid landscapes subject native plants to severe abiotic stresses, including salinity, drought, intense solar radiation, and high temperatures, along with biotic challenges such as herbivory, microbial pathogens, and competition. The ways in which plants coordinate responses to these simultaneous pressures remain insufficiently understood, creating a knowledge gap in desert persistence strategies. This study investigates the integrated defence mechanisms that enable survival under dual stress conditions. We analyse chemical responses such as the synthesis of antimicrobial and phenolic compounds, structural adaptations including thickened cuticles, trichomes, and reinforced cell walls, and physiological trade-offs affecting water-use efficiency and gas exchange. Emphasis is placed on the regulatory role of abscisic acid, which links abiotic stress responses to enhanced pathogen resistance through interconnected biochemical pathways. The study also evaluates the benefits and costs of these structural and biochemical defences. Our findings reveal that native desert species employ adaptive strategies that integrate structural, physiological, and biochemical processes to withstand simultaneous abiotic and biotic pressures. These coordinated mechanisms enhance plant persistence under extreme conditions and play a crucial role in maintaining biodiversity, ecological resilience, and the long-term stability of Qatar’s fragile desert ecosystems.

## 1. Introduction

**Figure 1:**
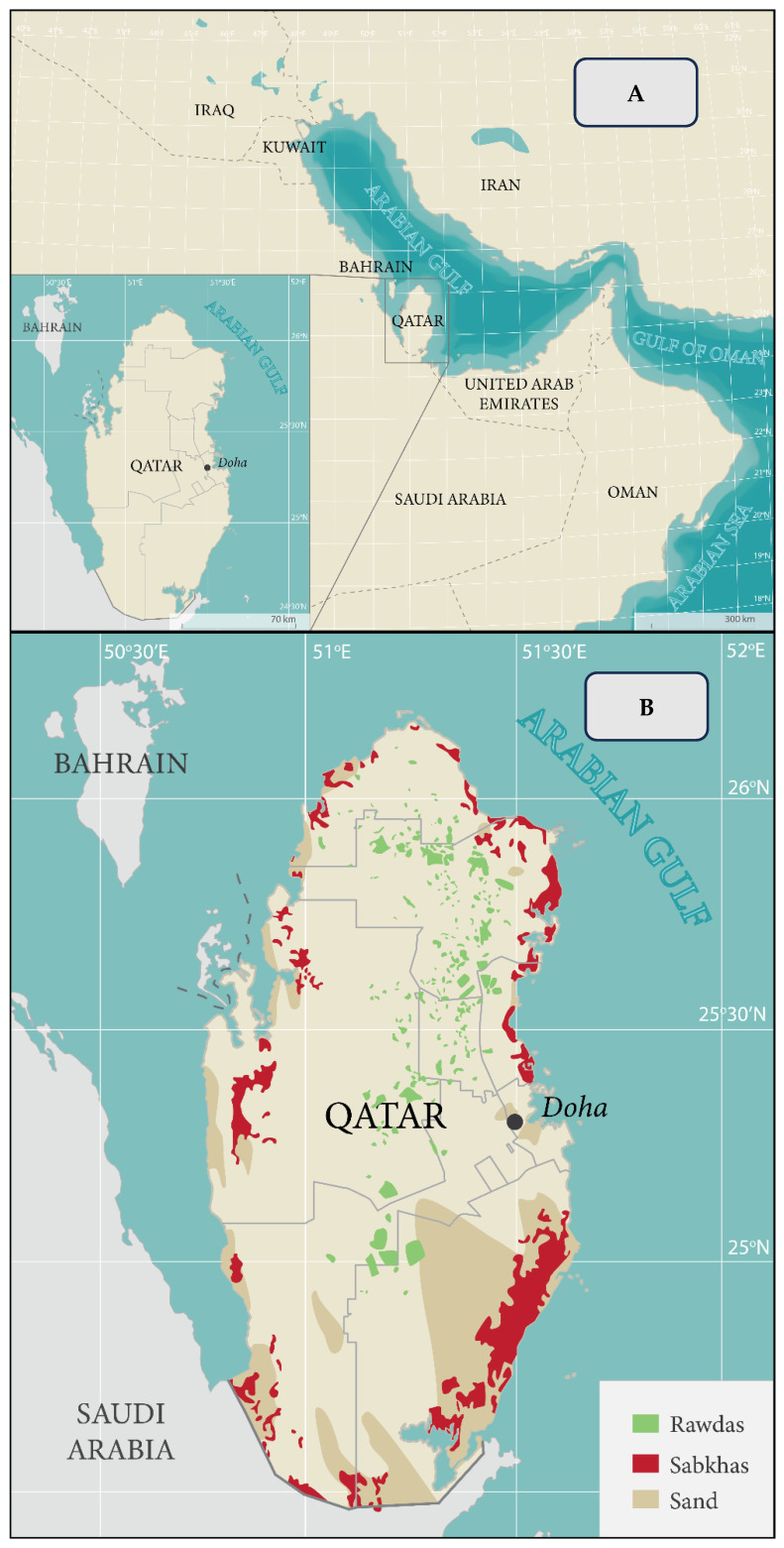
Map of the Arabian Gulf region and Qatar (**A**), and detailed map of Qatar (**B**) showing sabkhas (red patches), rawdhas (green patches), desert lands, saline soils (remaining areas), and the surrounding seawater of the Arabian Gulf.

The Arabian Gulf exhibits high salinity, extreme temperatures, and primarily arid to semi-arid landscapes, posing significant challenges to both crop cultivation and the survival of native plant species (Figure 1). In Qatar, soils found in sabkhas and coastal regions are particularly saline, with the electrical conductivity (ECe) of saturated soil extracts often surpassing 200 dS/m. The region experiences intense summer heat, with temperatures regularly exceeding 50 °C. The climate in Qatar is classified as arid to semi-arid, receiving an average annual precipitation of approximately 80 mm, which rarely surpasses 152 mm [1].

In such a severe environment, few wild plants, and animals can thrive. These native plants have developed various adaptations to endure the extreme conditions. Additional information is provided in a review by Yasseen and Al-Thani [2] who identified several xerophytic and halophytic genera as the primary inhabitants of the Qatari ecosystem. Many plants along the coastline and throughout Qatar are characterised as xerophytes, including species such as *Cyperus conglomeratus*, *Helianthemum lipii*, *Ochradenus baccatus*, *Oligomeris linifolia*, and *Tetraena qatarensis*. Halophytes, on the other hand, encompass approximately 26 genera reported in various studies and monographs, such as *Anabasis*, *Arthrocnemum*, *Atriplex*, *Avicennia*, *Halocnemum*, *Halopeplis*, *Limonium*, *Salsola*, *Seidlitzia*, and *Suaeda*. These plants employ three primary mechanisms to cope with dry and saline environments: (a) drought-escaping, (b) avoidance, and (c) tolerance mechanisms [2,3,4,5,6]. Table 1 shows the classification of these native plants and their main characteristics as reported by Yasseen and Al-Thani [2]. Notably, xerophytes are classified into three main groups, while halophytes were recognized into two main groups. Native plants in these regions may possess integrated mechanisms that coordinate responses to abiotic stresses—including structural modifications, physiological adaptations, and biochemical pathway alterations—with biotic defense strategies, such as resistance to microbial invasion, pathogen attack, and herbivory. In harsh ecosystems, such as those in the Arabian Gulf region, plants face extreme environmental conditions, including high salinity, drought, and elevated temperatures, as well as biotic pressures from pests and pathogens. These combined stresses require adaptive responses involving structural, physiological, and biochemical modifications. Such adaptations enable plants to maintain functionality and enhance survival under these challenging conditions [7].

Notably, biotic factors encompass all living components influencing an ecosystem’s structure, function, and dynamics. These include plants (such as weeds), animals (including insects, pests, nematodes, and herbivores that consume plants), as well as pathogens like bacteria, fungi, viruses, and other microorganisms. Broadly, biotic factors involve diverse interactions among organisms, including predation, herbivory, competition, symbiosis, parasitism, and decomposition. Although this article does not cover the full range of biotic interactions, it focuses on specific aspects relevant to Qatar’s native flora. It examines the methods and strategies adopted by native plant species to develop resistance against herbivory and microbial threats, highlighting how these plants survive and adapt to Qatar’s challenging environmental conditions [7,8].

**Table 1:** The main features and mechanisms of Qatari native plants in response to drought and salt Stress.

Xerophytes			
Variables	Features & Mechanisms	Species	References
Drought-escaping	These plants germinate, grow, and flower within a short period after heavy rainfall, and the seeds they produce remain dormant during the dry season	About 20 genera (28 species)	[2,9,10]
Drought avoidance	These adaptations are largely morphological and anatomical in nature. Two secondary mechanisms are involved: water conservation and accelerated water absorption (water spenders). Further details can be found in the literature	Many species such as *Aeluropus* lagopoides, *Cyperus conglomeratus*, *Euphorbia granulate*, *Mesembryanthemum nodiflorum Oligomeris linifolia*, *Portulaca oleracea*, *Polycarpaea spicata*, *Senecio desfontainei*	[2]
Drought tolerance	Osmotic adjustment is achieved through the accumulation of considerable amounts of organic and inorganic solutes—such as proline, glycine betaine, sugars, and inorganic ions—to enable tolerance to severe soil water deficit	Species such as *Ochradenus baccatus*, and *T*. *qatarensis*	[2]
**Halophytes**			
**Variables**	**Features & Mechanisms**	**Species**	**References**
Salt avoidance: salt exclusion, Salt extrusion, and Salt dilution	The mechanisms comprise structural and physiological adaptations that reduce cellular salt accumulation or promote the exclusion of salts through root membrane processes.	*Phoenix dactylefera*, *Limonium axillare*, *Avicennia marina*, *Atriplex* spp., *A*. lagopoides, *Tamarix* spp. *Halopeplis perfoliate*, *Suaeda aegyptiaca*	[2,5]
Salt tolerance	Osmotic adjustment has been considered as a main secondary mechanism to tolerate salt stress. In these plants, salt tolerance increases in both inorganic ions and organic solutes	Many crop and native plant species display these mechanisms to varying extents. A single plant species rarely depends on one mechanism alone; instead, several mechanisms often function in combination.	[2,5]

Additionally, microorganisms possess various means to infiltrate plant tissues, including natural openings such as stomata and lenticels, as well as wounds from external factors like soil particles, pathogenic attacks, or abiotic stresses, such as salinity, drought, and extreme temperatures [11]. Certain small animals within the ecosystem may also act as vectors, directly introducing microbes into plant interiors. Once inside, these microorganisms can colonize and migrate to different plant organs, including flowers, fruits, and seeds. Ultimately, some microbes, notably fungi and bacteria, can be transmitted to the next generation via seeds [12].

Native plants in Qatar display a range of morphological and anatomical adaptations that enable survival in the country’s extreme desert environment. These adaptations encompass specific forms, leaf orientations, and surface structures such as hairs and trichomes. Many desert plants, for example, have reduced leaf sizes or needle-like leaves to minimise water loss through transpiration [5,7]. *T*. *qatarensis*, a succulent common in Qatar’s arid regions, features fleshy leaves that store water and reduce evaporation. Similarly, *Haloxylon salicornicum*, possesses vertically oriented stems, which lessen direct exposure to intense sunlight and conserve moisture.

Trichomes, small hair-like structures on plant surfaces, are prevalent in desert flora and serve multiple protective functions. They can reflect sunlight, reduce leaf temperature, and act as barriers against insects and excessive wind, which can desiccate plant tissues. In Qatar, species such as *L*. *axillare* exhibit these traits, supporting their survival in sandy, saline, and extremely hot conditions. Beyond physical adaptations, many native plants produce specific chemical compounds serving as biochemical defences. These compounds deter herbivores, inhibit pathogen growth, and eliminate invading organisms. *Calligonum comosum*, a desert shrub found in Qatar, produces antimicrobial secondary metabolites that protect against bacterial and fungal infections. Additionally, the presence of phenolic compounds, alkaloids, and essential oils in various species enhances resistance to pests and microbial invasion. These chemical defences are crucial in an ecosystem where biological threats are intensified due to limited resources and competitive pressures. Together, these physical and chemical adaptations allow Qatar’s native plants to endure extreme heat, drought, salinity, and biotic stress, making them vital components of the country’s unique desert ecosystem [8,13].

This article investigates the physical and chemical defence mechanisms, along with symbiotic relationships, that enable native plants in Qatar to withstand biotic stresses and mitigate potential threats. In addition to these defences, native plants employ competitive strategies to secure essential resources such as sunlight, water, and nutrients. With approximately 400 native plant species documented in Qatar [14], the region provides a valuable setting for exploring desert flora. Notably, about 40% of these species are range plants with high nutritional value, containing proteins, carbohydrates, fatty acids, essential elements, and other bioactive compounds with potential pharmaceutical, nutritional, cosmetic, and economic applications [15]. Despite increasing global interest in plant adaptation to extreme environments, comprehensive reviews integrating the biotic defence mechanisms of native desert species in Qatar remain limited. Existing studies often focus on either abiotic stress or isolated plant–pathogen interactions, leaving a gap in understanding how multiple stressors jointly shape plant survival strategies. This review addresses that gap by synthesizing current knowledge on the structural, physiological, and biochemical defences of Qatar’s native flora, highlighting their ecological significance and potential practical applications. By providing an updated synthesis, this work aims to advance understanding of plant resilience in arid ecosystems and support future research on sustainable biodiversity management in the region.

## 2. Mechanisms of Resistance Against Biotic Stress

Biotic stresses can damage plant tissues, reduce photosynthesis, stunt growth, and ultimately lower crop yield and quality. In response, plants employ various defense mechanisms, such as altering physical structures, producing toxic chemicals, or activating immune responses. Notably, solute accumulation under stress conditions may serve as a pre-emptive measure, enabling plants to cope with both environmental challenges and opportunistic microbial threats. Microorganisms, including bacteria, fungi, algae, and protozoa, commonly colonize different plant organs [16]. Plants offer a variety of microhabitats, including the rhizosphere (root-influenced zone), the phyllosphere (aerial parts), and the endosphere (internal transport system). Interactions between microorganisms and plants can be beneficial or detrimental, categorized as neutralism, commensalism, synergism, mutualism, amensalism, competition, or parasitism [2,17].

Plants synthesise a diverse array of chemical compounds crucial for defence against various environmental threats. The biosynthesis of these compounds often intensifies when plants face abiotic stresses such as salinity, drought, and extreme temperatures [6]. Early research indicates that these stresses induce structural and biochemical changes at multiple levels, including morphological, anatomical, physiological, and molecular aspects. For example, in salt-affected wheat plants, mesophyll cells become more compact, intercellular spaces and vascular elements decrease in size, and the cuticle thickens [18]. Additional changes include an increased number of mesophyll layers and lobes within the mesophyll cells [19,20,21].

Subsequent studies have demonstrated that abiotic stress factors significantly affect structural attributes, such as cell shape and area, ultimately altering overall cell volume [2,22]. Furthermore, stress conditions lead to notable changes in the chemical composition of external protective structures and internal membrane systems [18]. These modifications enhance plant adaptation to adverse environments, improve resistance to microbial pathogens, and strengthen responses to both biotic and abiotic stresses [23]. At the molecular level, these adaptive responses involve altered gene expression, protein modifications, and the restructuring of cellular components. Together, these changes influence plant growth, development, and survival under challenging conditions [24,25,26].

Native plants, including xerophytes, halophytes, and hydrophytes, have evolved a diverse range of strategies and mechanisms to cope with environmental challenges. These adaptations occur at behavioral, morphological, physiological, and biochemical levels, enabling plants to withstand both abiotic and biotic pressures [2,4,6,16,27]. In Qatar, native flora have developed specialised mechanisms to counteract biotic stressors such as herbivory, pathogen attacks, and competition from neighbouring plants. These adaptations ensure their survival and persistence in the region’s harsh, arid ecosystems [28]. These mechanisms can be broadly categorised into (a) physical defences, (b) chemical defences, and (c) symbiotic associations. Concurrently, advancements in biotechnology over recent decades have led to the development of transgenic plants with enhanced resistance to biotic stresses. These advances provide valuable insights and complementary approaches to the natural defence strategies observed in native species [29,30,31].

### 2.1. Physical Defences

Physical defences are essential strategies that plants use to mitigate herbivory and pathogen attacks. Many plants produce thorns and spines, which act as mechanical deterrents by injuring or discouraging herbivores from feeding on stems, leaves, or reproductive structures [32]. Trichomes, or leaf hairs, not only serve as barriers that prevent herbivores from easily accessing the plant surface but also impede insect movement and oviposition, thereby reducing herbivore success [33]. Moreover, waxy leaf coatings create a protective layer that minimises water loss and forms a slippery surface, deterring insects from feeding efficiently; these coatings can also obstruct colonisation by microorganisms such as fungi and bacteria [34]. Thick bark and cuticles provide robust physical shields that reduce penetration by pests and pathogens and limit environmental stressors like desiccation and UV damage [35]. These structural adaptations collectively demonstrate the multifaceted methods plants use to employ physical barriers for enhanced survival, a subject that will be explored in greater detail throughout this article. Some examples from the Qatari habitats have been discussed in Section 3.1.

### 2.2. Chemical Defences

Plants utilise diverse defensive strategies, such as producing toxins and allelochemicals and releasing volatile compounds, to protect against predators, insects, and microorganisms. These substances deter herbivores and pathogens by rendering the plant unpalatable or harmful or by directly inhibiting or eliminating harmful organisms, such as fungi [31]. For instance, chrysanthemums produce pyrethrin, a natural neurotoxin [36,37] that produced by *Chrysanthemum* spp. These species include *C. coronarium* and others that reported by Rizk and his collaborators [37,38], while mint and witch hazel release antibacterial compounds [39].

Several studies have identified various toxic proteins in plant parts, including roots, tubers, stems, fruits, buds, and foliage. These include ribosome-inactivating proteins, lectins, protease inhibitors, α-amylase inhibitors, canatoxin-like proteins, ureases, arcelins, antimicrobial peptides, and pore-forming toxins. These compounds exhibit significant biological activity and have potential applications in crop protection, drug development, cancer therapy, and genetic engineering. Despite their toxicity, these proteins are valuable bioactive molecules due to their ability to interfere with specific physiological and cellular processes. However, they can also pose health risks to humans and animals, as detailed by Kocyigit et al. [40].

Advancements in molecular biology and biotechnology have further elucidated the cellular mechanisms of these natural compounds. For example, plant defence responses are activated through complex biochemical pathways often regulated by various transcription factors [41]. Native plants produce toxins through several methods:

(a) Defence against herbivores: Some plants produce toxins that poison herbivores, while others generate compounds that interfere with growth and digestion. *Phacelia* plants (Family: Boraginaceae), for example, have trichomes containing poisons, insecticides, and allergens. Many examples and details from the Qatari flora were given by Rizk and others [37,38].
(b) Resistance to pathogens: Certain plants produce toxins to combat pathogens such as bacteria and fungi. These toxins disrupt metabolic pathways and damage cell structures, preventing infection [30].
(c) Defence against environmental stressors: Some plants produce toxins in response to extreme environmental conditions. These toxins help by reducing water loss and protecting internal structures and organelles from damage by reactive oxygen species (ROS) [42].

Plant toxins include various compounds, such as alkaloids, which are poisonous to animals; glycosides, like cyanogenic glycosides, which release toxins upon tissue damage; resins, which may cause irritation or possess toxic properties; and tannins, which interfere with herbivore digestion.

### 2.3. Symbiotic Relationships and Ecological Niches

A symbiotic association is a close, long-term interaction between two different organisms, where at least one partner benefits. In plants, such associations may involve fungi, bacteria, other plants, or even animals, and can range from beneficial to neutral or harmful. Interactions with microorganisms are particularly significant, as higher plants are colonised by diverse microbial groups that occupy different ecological niches. These include the rhizosphere, the soil region influenced by roots; the phyllosphere, the aerial surfaces of the plant; and endophytes, which inhabit internal plant tissues without causing disease. Endophytic associations are often considered true symbioses, as they frequently enhance plant stress tolerance, growth, and pathogen resistance, though some may be commensal. Overall, plant-microbe interactions span a spectrum of outcomes and are commonly classified as neutralism, commensalism, synergism, mutualism, amensalism, competition, or parasitism [17]. Among these, parasitism represents a form of association where one organism benefits at the expense of the other. Recent work by Fahmy and Al-Thani [43] illustrates this by showing that the holoparasitic plant *Cynomorium coccineum* effectively exploits its host, *T*. *qatarensis*, by extracting both water and nutrients.

Plant–microorganism symbioses improve tolerance to salinity and water stress through coordinated physiological, biochemical, and molecular mechanisms. In halophytes, microbes such as *Bacillus* spp. and *Pseudomonas* spp. function as endophytes, facilitating soil desalination and phytoremediation [6]. In the Qatari sabkhas, bacteria and fungi inhabiting both the rhizosphere and endosphere further enhance halophyte resistance to salinity and other stressors, including hydrocarbon pollution. These associations induce the production of bioactive compounds, demonstrating positive biotic interactions that bolster the resilience of native plants in extreme conditions. Recent studies provide additional details [6,44,45,46,47,48,49,50]. Leveraging these interactions, especially in saline or degraded soils, represents a promising strategy for sustainable agriculture [51].

The following methods elucidate these relationships: nutrient and water uptake by arbuscular mycorrhizal fungi (AMF) and plant growth-promoting rhizobacteria (PGPR) are key biological methods that enhance plant nutrition and water status. AMF improve nutrient and water uptake by physically extending the root system via hyphal networks, while PGPR contribute through biochemical interactions, such as the production of phytohormones and enzymes. Together, these symbionts strengthen plant stress-tolerance mechanisms. The use of AMF and PGPR as bio-inoculants holds significant potential for increasing crop productivity, particularly in challenging environments like saline or nutrient-depleted soils [52].

AMF form symbiotic relationships with the roots of most terrestrial plants. They penetrate the root cortical cells, creating structures such as arbuscules and vesicles that facilitate nutrient exchange. Enhanced nutrient uptake includes phosphorus mobilisation achieved by hyphal networks extending into the soil beyond root zones, thereby aiding the uptake of micronutrients such as copper, iron, and zinc [53]. Additionally, hyphal networks extend the root system, improving water status under conditions of salinity and drought. Furthermore, AMF reduce oxidative stress by enhancing antioxidant enzyme activities in plants and improve osmotic adjustment and root hydraulic conductivity. PGPR are beneficial bacteria that colonise the rhizosphere and promote plant growth through direct and indirect mechanisms [54].

PGPR are beneficial bacteria associated with the root system in the rhizosphere that promote plant growth through both direct and indirect mechanisms. These mechanisms include nitrogen fixation, as certain PGPR fix atmospheric nitrogen [55], while others secrete organic acids and enzymes that solubilise insoluble phosphorus into absorbable forms for plants [56]. Furthermore, these microorganisms produce and modulate phytohormones such as auxins, cytokinins, and gibberellins, which stimulate root growth and enhance nutrient uptake, thereby increasing stress resistance [57]. PGPR also aid in stress alleviation by enhancing antioxidant defences and promoting the accumulation of compatible osmolytes [58]. These solutes, including proline, glycine betaine, and soluble sugars, help maintain cell turgor pressure and protect cellular structures under stress conditions [48,49,59]. Additionally, some strains produce and activate ACC deaminase, which reduces ethylene levels in stressed plants, thus promoting root development [60,61]. Both AMF and PGPR can enhance soil structure and water-holding capacity, which mitigates the effects of salinity on water availability for crops. Importantly, these microbes improve soil health, making it more resilient to salinity stress and more capable of retaining water for plants [62].

Many microbial species, known as endophytes, inhabit plant tissues and can enhance nutrient uptake and stress tolerance. Recent studies have examined the role of endophytes in boosting the remediation potential of halophytes—salt-tolerant plants such as *H*. *perfoliata*, *Salicornia europaea*, *Salsola soda*, and *T. qatarensis*—in polluted soils and waters. The microorganisms associated with these halophytes may support the plants’ ability to remediate saline and contaminated environments. For further details, refer to Yasseen and Al-Thani [6], Al-Thani and Yasseen [63], and Al-Thani et al. [64].

AMF and PGPR frequently exhibit synergistic effects, enhancing root colonisation and increasing overall plant resilience. These combinations have been effective in remediating polluted soils affected by salt stress, petroleum hydrocarbons, and heavy metals.

### 2.4. Competition Between Native Plants in Qatar

Biotic interactions among plants for essential resources such as water, nutrients, and, occasionally, sunlight are crucial factors that influence plant growth and development. Al-Thani and Yasseen ([49], Figure 10) reported observations in Qatari sabkhas, where communities of *Halocnemum strobilaceum* display a distinctive pattern. In these communities, plants die in the centre, leaving behind bare patches of dead branches, while green growth persists at the periphery. Over time, this dieback expands outward toward the community’s edges. Such patterns underscore how competition and other plant-plant interactions function as biotic factors, shaping the distribution and survival of native species in saline and arid environments. Plants use various strategies to outcompete neighbouring species, such as rapid growth, efficient resource utilisation, and allelopathy—the release of chemicals that suppress competitors. In Qatar, native plants engage in diverse and complex interactions, including facilitation, allelopathy, and intense competition. Competition is particularly pronounced in arid and saline environments, where water and nutrients are severely limited. Although sunlight is usually abundant, it may become scarce under conditions of shading, dense vegetation, or physiological stress that limits a plant’s ability to utilise available light. Species like *Fagonia indica*, *Gisekia pharnacioides*, *Grewia erythraea*, and *Haloxylon salicornicum*, which also serve as important livestock fodder, exemplify the struggle for survival in such environments. Other species, such as *C*. *conglomeratus* and *H*. *lipii*, have specialised adaptations that allow them to efficiently absorb and retain water. While these traits improve the fitness of individual plants, they also heighten competition for scarce water resources, making water one of the most critical limiting factors in the Qatari desert ecosystem.

#### 2.4.1. Adaptive Strategies to Compete for Sunlight

Plants in Qatar, which thrive in extremely arid, saline, and nutrient-poor environments, have developed diverse adaptive strategies to compete for sunlight—an essential yet intense resource in desert ecosystems. These strategies include:

Growth form and canopy structure: *H*. *salicornicum* grows upright with minimal branching, reducing self-shading and allowing greater light penetration. This minimises competition among neighbouring plants. Conversely, *T*. *qatarensis* exhibits a low-growing form. Additionally, 16 species from 13 genera in Qatar feature rosette leaves [9,10]. These rosette forms lie close to the ground, capturing early morning and late afternoon light while minimising water loss. Most desert plants also maintain sparse canopy spacing to decrease direct competition for light. However, in microhabitats such as depressions or shaded areas near rocks, competition for light can intensify.

Leaf orientation and morphology: Many Qatari plants possess small or narrow leaves to minimise water loss while efficiently absorbing sunlight. For instance, *F*. *indica* features small, reduced leaves. Leaves may also orient vertically or at angles, avoiding intense midday sun and preventing overheating. This orientation allows plants to capture light during cooler hours while minimising shading of nearby plants.

Spacing strategies: Some species reduce competition for light through wide spacing, self-thinning, or competitive exclusion. *H*. *salicornicum* and *Panicum turgidum*, for example, grow in widely spaced clumps, ensuring unobstructed access to sunlight. This spacing also reduces competition for limited soil moisture and nutrients.

#### 2.4.2. Adaptive Strategies to Compete for Water

Many native plants in Qatar have evolved specific strategies to manage environmental stressors such as drought and soil salinity. Generally—and particularly in Qatar—plants employing these strategies either store water internally or absorb it efficiently from the soil. Several examples illustrate these adaptive strategies. *T. qatarensis*, a xero-halophytic species from the *Chenopodiaceae* family, stores water in its fleshy leaves and is classified as an inducible crassulacean acid metabolism (CAM) plant [6,65]. Other noteworthy members of the same family include *Arthrocnemum macrostachyum*, *Halocnemum strobilaceum*, *H*. *perfoliata*, and *S*. *aegyptiaca*. These species exhibit succulence, a trait that allows them to retain water and tolerate harsh desert and saline conditions [22].

Additionally, competition for limited water resources is a critical survival strategy among native plants in drought- and aridity-affected environments. For instance, *H*. *lipii* enhances water uptake by developing a high root-length density in deeper soil layers relative to its leaf area, enabling more effective moisture access. Similarly, *C*. *conglomeratus* exhibits a high root-to-shoot ratio, increasing the amount of water absorbed per unit of leaf area. This trait supports a drought avoidance mechanism, allowing the plant to survive under prolonged dry conditions [2,22].

#### 2.4.3. Adaptive Strategies to Compete for Nutrients

Soils in Qatar are generally deficient in major nutrients, particularly nitrogen and phosphorus [66,67], leading to intense competition among plants for limited resources. This scarcity, combined with the arid climate, high temperatures, and elevated evaporation rates, presents significant challenges for plant growth. In this context, microorganisms, particularly mycorrhizal fungi, play a crucial role in enhancing plant survival and resilience. AMF, such as *Glomus*, *Rhizophagus*, and *Acaulospora*, are commonly associated with native Qatari plants. These plant species were listed by Yasseen and Al-Thani [6], which include *A*. *marina*, *Cressa cretica*, *Heliotropium* spp., *L*. *axillare*, *Polypogon monspeliensis*, *Sporobolus* spp., *Suaeda* spp., *Tamarix* spp., and *Teucrium polium*. They enhance phosphorus uptake, water absorption, and tolerance to drought and salinity [68]. Other fungi, such as ectomycorrhizal fungi (ECM), including genera like *Pisolithus* and *Laccaria*, assist in nitrogen acquisition from organic sources and support plant growth in nutrient-poor soils [69]. These fungi form symbiotic networks with plant roots, effectively extending the root system and facilitating nutrient exchange, making them essential to plant survival in Qatar’s harsh, nutrient-deficient environments. Additionally, these fungi not only improve nutrient and water uptake but also increase plant tolerance to drought and salinity, reduce pathogen infections, and contribute to soil structure and fertility.

## 3. Native Plants of Qatar and Their Biotic Challenges

As sessile organisms, plants are particularly susceptible to environmental and biotic stressors. To endure these pressures, they have evolved various defence mechanisms that allow either tolerance or avoidance of such challenges. These mechanisms encompass chemical defences and structural modifications that limit pathogen colonisation and deter herbivory [33,70,71]. The native flora of Qatar exhibits diverse adaptive traits that mitigate the effects of herbivory, pathogens, and interspecific competition, while also conferring resilience to the country’s extreme abiotic conditions, such as high salinity, prolonged drought, elevated temperatures, and intense solar radiation [2,7,72].

### 3.1. Qatari Native Plants Resist Herbivores

Livestock such as camels, goats, sheep, and gazelles are unable to consume certain native plants due to the evolution of defensive traits in these plants, such as thorns, tough leaves, bitter sap, and toxic chemicals—including alkaloid compounds [38,73]. Consequently, many Qatari native plants, including *Acacia tortilis*, *Aerva javanica*, *Calotropis procera*, *Cornulaca monocantha*, *Haloxylon salicornicum*, *Leptadenia pyrotechnica*, *Nerium oleander*, *Ricinus communis*, *Salsola imbricata*, and *T. qatarensis*, are inedible to these animals. Conflicting reports exist regarding the edibility of certain native species for domestic livestock. This section outlines selected plant species native to Qatar.

For instance, the ingestion of *Acacia tortilis* by cattle has been associated with undesirable sensory alterations in milk, such as off-odours. *Aerva javanica* has morphological adaptations, including hairy stems and leaves, which reduce its palatability and deter grazing by most livestock. Despite this, it is readily consumed by small ruminants, particularly goats and sheep, while larger herbivores, such as camels and cattle, may also utilise it as forage, especially during drought conditions when alternative feed resources are scarce. *Calotropis procera*, noted for its large leaves and purple flowers, produces a toxic milky latex harmful to humans and livestock. Although toxic, it has long been used in traditional medicine. In contrast, *Acacia tortilis* is protected from over-browsing by its sharp thorns but remains an important forage resource in arid and semi-arid ecosystems, including the Arabian Gulf region. Its pods are particularly high in crude protein, enhancing milk production and providing a significant nutrient source for camels and goats. At the same time, the ingestion of *C. procera* can cause serious effects such as gastrointestinal upset and cardiotoxicity.

*Cornulaca monocantha* is a resilient shrub characterised by spiny stems and leaves, making it well-adapted to arid environments. It is largely unpalatable due to its bitter taste, which discourages grazing, though it is reported to have certain traditional medicinal applications. *H. salicornicum*, a hardy shrub with tough stems and low palatability, is common in sandy soils. It resists grazing and remains an important fodder resource in arid regions, staying green during prolonged dry periods and thus providing vital forage when alternative vegetation is scarce.

*Leptadenia pyrotechnica* features tough stems and adaptations that confer resistance to herbivory, such as the secretion of a bitter, toxic latex rich in diverse chemical constituents, including secondary metabolites as chemical defences. To date, 104 compounds from various phytochemical classes have been identified in this species. Pharmacological studies reveal notable diuretic activity at concentrations ranging from 10 to 300 mg/kg, with doses of 100 and 300 mg/kg producing significant diuretic and saluretic effects comparable to those of furosemide. Leaf extracts have demonstrated diuretic efficacy in both acute and prolonged experimental models, likely mediated through mechanisms involving carbonic anhydrase inhibition, prostaglandin modulation, and cholinergic pathway activation.

*Nerium oleander* is a woody evergreen shrub with leathery, lance-shaped leaves and colourful flowers. It is drought-tolerant and frequently cultivated in gardens. All parts of the plant are highly toxic due to cardiac glycosides, posing serious risks to humans and livestock. Traditionally, it has been used medicinally for cancer, uterine stimulation, malaria, dropsy, and skin conditions, but incorrect dosage can cause central nervous system (CNS) depression and other toxic effects. Moreover, the plant demonstrates potential for phytoremediation of pollutants.

*Ricinus communis* (castor oil plant) is widely cultivated as both an ornamental species and a source of valuable oil, particularly in irrigated fields and garden soils. The plant shows considerable potential for phytoremediation of heavy metals and organic pollutants [63,74,75,76]. *R. communis* preferentially accumulates heavy metals such as cadmium (Cd), manganese (Mn), nickel (Ni), and lead (Pb) in its leaves, while vanadium (V) concentrates in the roots. These metals can reach toxic levels when their concentrations in the growth medium exceed specific thresholds [77,78]. Additionally, *R. communis* effectively degrades and removes persistent organic pollutants (POPs), including hexachlorocyclohexane (HCH), DDT, heptachlor, and aldrin. Bauddh et al. [79] highlighted *R. communis* as a promising non-edible phytoremediator, noting its resilience and multipurpose utility as an industrially important, oil-yielding shrub. Recent studies indicate that microorganisms associated with *R. communis* may significantly enhance both bioremediation and phytoremediation processes [48,49,59].

*Salsola imbricata* has densely pubescent leaves and salt-accumulating tissues that confer resistance to herbivory. The species exhibits notable antioxidant and antimicrobial activities, mainly due to its complex phytochemical profile. Aerial extracts are rich in secondary metabolites, including phenolic acids, fatty acids, and steroids, all contributing to their bioactivity. In vitro antimicrobial assays reveal significant inhibitory effects against multiple bacterial and fungal strains, with activity levels varying according to extract concentration. These findings highlight the potential of *S. imbricata* extracts as natural agents in agricultural applications and warrant further investigation into their mechanisms of action and practical efficacy.

*Tetraena qatarensis* is a halophytic and drought-tolerant plant with leathery leaves that synthesises a diverse array of secondary metabolites both endogenously and through associated endophytic microorganisms. These metabolites include alkaloids, terpenoids, and flavonoids, many of which serve as chemical defences by deterring herbivores or exerting toxicity towards them. Additionally, certain endophytic fungi and bacteria produce bioactive compounds with antifungal and antibacterial properties, potentially impacting herbivores by interfering with their digestive processes. The combined action of plant- and microbe-derived metabolites illustrates the multifaceted chemical defence strategies employed by *T. qatarensis*, underscoring its ecological resilience and potential for bioactive compound exploration.

Over the past four decades, a substantial body of research, including numerous studies and monographs, has been published on the chemical constituents and phytochemistry of various macroalgae, cyanobacteria, and native plants, including rangeland species, poisonous plants, and horticultural varieties. Readers are encouraged to consult these monographs for more detailed information [15,37,38,80,81].

### 3.2. Qatari Native Plants Resist Pathogens

Wild plants in Qatar have developed numerous mechanisms to either avoid or tolerate abiotic stressors, as highlighted in several studies [2,5,6]. These strategies may also enhance resistance to biotic factors, including living organisms such as microorganisms and pathogens. This resistance is achieved by inducing changes in the chemical composition of both internal and external plant structures. External structures, such as the cuticle, natural openings like stomata, and wounds, potentially provide pathways for pathogens to access the plants’ internal compartments.

#### 3.2.1. The Role of the Cuticle

The structure and function of cuticle have been described in detail in many reports and monographs [82]. However, a recent review by Arya et al. [83] worth to mentioned here. The epidermis, the outermost cellular layer of plants, is enveloped by a protective cuticle primarily composed of cutin, a waxy and hydrophobic biopolymer. This cuticular layer plays a crucial role in minimizing transpirational water loss and serves as a frontline defense against pathogenic invasion. Structurally, cutin is a cross-linked polyester mainly synthesized from C16 and C18 hydroxy fatty acids, which may possess up to three hydroxyl functional groups. Representative monomers include 16-hydroxypalmitic acid, 9,16-dihydroxypalmitic acid, and 10,16-dihydroxypalmitic acid in the C16 series, while the C18 group typically comprises 18-hydroxyoleic acid, 9,10-epoxy-18-hydroxystearic acid, and 9,10,18-trihydroxystearic acid. The inherent hydrophobicity and resistance of cutin to enzymatic degradation contribute to its efficiency as a protective barrier, promoting water conservation and enhancing plant resilience to both abiotic and biotic stressors. Additionally, the cuticle functions as a mechanical barrier, restricting the penetration of microorganisms into internal plant tissues and supporting the overall structural integrity of the epidermis.

Native plant species inhabiting the arid and saline environments of Qatar and other regions of the Arabian Gulf have evolved distinct morphological, anatomical, physiological, and biochemical traits that enable them to survive and reproduce under extreme conditions. An early study by Abulfatih [84] described the morphological and anatomical adaptations of several native xerophytic and halophytic species, including *Calotropis procera*, *C*. *conglomeratus*, *Fagonia ovalifolia*, *Glossonema varians* (syn. *Glossonema edule*), *Heliotropium bacciferum*, *Lycium shawii*, *O*. *baccatus*, *Sporobolus iocladus* (syn. *Sporobolus arabicus*), *Tamarix ramosissima*, *Vachellia flava* (syn. *Acacia ehrenbergiana*), and *Ziziphus nummularia* (syn. *Rhamnus nummularia*). These species exhibit morphological and anatomical features that reflect advanced adaptations to Qatar’s harsh habitats. Prominent among these is the development of a thick cuticle, along with well-developed mechanical and supportive tissues such as vascular bundles, fibers, and sclereids.

The thick cuticle observed in these native species provides a multifaceted defense against environmental stressors, including extreme temperatures, high solar radiation, and limited water availability. According to Yasseen and Al-Thani [2], *C*. *conglomeratus* and *T*. *qatarensis* possess extensive root systems that enhance water uptake, while their robust cuticular layers reduce water loss and prevent pathogen entry. In addition to its physical properties, the cuticle may contribute to chemical defense, as some plants synthesize and deposit antimicrobial compounds within it that inhibit pathogen growth [31,85,86]. These structural and biochemical adaptations collectively enhance plant survival, enabling native species to maintain physiological function and ecological persistence under the arid and saline conditions characteristic of the Arabian Peninsula.

Early studies on wheat plants [18] clearly show that plants exposed to salinity have thicker cuticles. This thick cuticle plays a crucial role in several aspects: (1) Xerophytes and halophytes possess thick cuticles as a strategy to protect against water loss through transpiration, thereby conserving water under drought and salinity conditions. (2) The cuticle acts as a physical barrier, protecting the plant from excessive salt entry through the epidermis, which can damage the internal structures of cell organelles. (3) Chemically specialised, the thick cuticle withstands extreme environmental conditions such as intense heat, water scarcity, high radiation—including UV—and microbial attacks, particularly through tissue damage. (4) A thick cuticle generally signifies a high wax load, including long-chain alkanes, fatty alcohols, and esters, and contains higher proportions of triterpenoids and phenolic compounds. (5) These compounds are antimicrobial and enhance the cuticle’s hydrophobicity, thereby reducing transpiration. (6) Salt crystals on the leaf surface play vital roles in halophytes, including excess salt removal, leaf surface cooling, maintaining osmotic balance for water flow from the soil to aerial parts, and providing antimicrobial protection. (7) Stomatal behaviour is a critical strategy restricting microbial entry into plant tissues. While the thick cuticle serves as a physical barrier against pathogens, partial or complete stomatal closure adds an additional defence layer by limiting pathogen access through natural openings. However, the internal tissues of desert plants and halophytes often remain vulnerable, as drought and salt stress can impair immune responses by redirecting resources away from defence mechanisms, thus increasing susceptibility to infection. Interestingly, stress-induced modifications like cuticle thickening and stomatal closure, primarily adaptations to abiotic stress, also reduce pathogen entry] [87,88,89]. Consequently, stomatal closure functions not only as a key adaptation to abiotic stresses such as drought and salinity but also as a critical defence against microbial invasion, acting as a “door-closing” strategy that restricts pathogen entry through the pores. Nonetheless, this protective role may be compromised when environmental stresses weaken the plant’s immune system, thereby increasing disease susceptibility in desert plants and halophytes [90,91].

In contrast, mesophytic plants typically thrive in temperate, moist environments and have a less chemically fortified cuticle. This group includes most crop species—such as wheat, corn, beans, and tomatoes—as well as various garden and forest plants, including deciduous trees and grasses. These plants generally have a thinner cuticle with lower wax content. The waxes they produce usually exhibit simpler chemical profiles, often composed of shorter-chain hydrocarbons. Mesophytes also contain fewer compounds associated with antimicrobial activity or UV protection, and their overall surface hydrophobicity is lower since water conservation is less critical in their native habitats. Table 2 compares desert plants with mesophytes (non-stressed plants), highlighting key differences in the structural and chemical characteristics of their outer protective layers.

Environmental stress conditions can induce significant physiological and biochemical responses in native wild plants of Qatar, bolstering their defences against both abiotic and biotic factors. Drought and salinity, for instance, create osmotic and ionic imbalances that trigger a cascade of stress responses at cellular and molecular levels. A key adaptation involves enhancing or modifying cutin biosynthesis, the process that forms the cutin polymer contributing to the plant’s outer cuticle. These modifications can strengthen the cuticle barrier, reduce water loss, and increase resistance to environmental stresses and pathogen invasion. The potential mechanisms include: (1) an increase in cuticular lipid content, (2) thickening of the cuticle, (3) elevated gene expression related to cutin biosynthesis, (4) negative impacts on photosynthesis, (5) activation of antioxidants, and (6) membrane remodeling.

#### 3.2.2. Key Pathways and Enzymes Involved in Cutin Biosynthesis

Kolattukudy and his colleagues’ early work offered foundational insights into the biosynthesis of wax and cutin in plant cuticles [101,102]. Many authors have further elaborated on the biosynthesis of wax components. Scientists and students interested in this topic are encouraged to review relevant studies and monographs for additional details [103,104,105]. Two primary groups of enzymes play direct roles in cutin biosynthesis, while three other groups, which include enzymes, proteins, and genes, may contribute indirectly, particularly under severe environmental conditions.

(A) Glycerol-3-phosphate acyltransferases (GPATs) are essential enzymes in the synthesis of cutin. They catalyse the acylation of glycerol-3-phosphate (G3P) by esterifying acyl groups at the sn-2-position of G3P.

$$\mathrm{Glycerol}\text{-}3\text{-}\mathrm{phosphate}+\mathrm{Acyl}\text{-}\mathrm{CoA}\overset{\mathrm{GPAT}}{\to }\mathrm{Lysophosphatidic}\;\mathrm{acid}\;\left(\mathrm{LPA}\right)+\mathrm{CoA}$$

GPAT (glycerol-3-phosphate acyltransferase) exists in multiple isoforms, some of which are directly involved in cutin biosynthesis. When the GPAT catalysed reaction is followed by dephosphorylation via a phosphatase, it produces sn-2 monoacylglycerol (2-MAG), a key intermediate in this pathway [106]. In *Arabidopsis*, specific isoforms such as GPAT4, GPAT6, and GPAT8 catalyse the acylation of glycerol-3-phosphate or 2-MAG with ω-hydroxy fatty acids, which are precursors for cutin monomers. Sui et al. [107] demonstrated that salt stress induces the expression and activity of GPAT in the halophyte *Suaeda salsa*, a species found in the flora of Qatar. GPAT is crucial in helping the plant adjust its membrane lipid composition to withstand the detrimental effects of salinity. When the gene encoding this enzyme is expressed in *Arabidopsis*, it enhances physiological functions, including membrane stability, water retention, and salt tolerance. These findings confirm the enzyme’s significant role in improving plant resilience under saline conditions. Beyond its role in stress responses, GPAT is involved in the biosynthesis of cutin—a polyester forming part of the plant cuticle on aerial surfaces—where it contributes to pathogen defence, environmental stress resistance, and maintenance of organ integrity.

(B) Cytochrome P450 Monooxygenases (CYP86 and CYP77 Families): Environmental stresses, including salinity and water deficit, significantly influence the activity of cytochrome P450 (CYP) monooxygenases. These enzyme families are integral to cutin biosynthesis in plants. Specific members, such as CYP709B3, are also involved in abscisic acid (ABA) biosynthesis, which regulates stomatal movement and facilitates plant responses under salt stress [108]. The role of cytochrome P450 monooxygenases in cutin biosynthesis is crucial for enhancing salt resistance, as cutin forms a protective barrier that reduces water loss. These enzymes participate in various plant growth and developmental processes, helping mitigate a range of stress conditions [109]. The following equations illustrate the initial steps in the biosynthesis of hydroxy fatty acids, which serve as precursors for cutin monomers.

$$\begin{array}{c} {\mathrm{Fatty}\;\mathrm{acid}+\mathrm{NADPH}+O}_{2}\to \omega \text{-}\mathrm{Hydroxy}\;\mathrm{fatty}\;{\mathrm{acid}}^{*}+{\mathrm{NADP}}^{+}+H_{2}O\\ {\,}^{*}\;\mathrm{Or}\;\mathrm{mid}\text{-}\mathrm{chain}\;\mathrm{hydroxy}\;\mathrm{fatty}\;\mathrm{acid}. \end{array}$$

Hydroxylated fatty acids are subsequently polymerised to form cutin, a key structural component of the plant cuticle. Studies have demonstrated that salt stress upregulates the activity and expression of CYP86 and CYP77 family members (Cytochrome P450, families 86 and 77, respectively) as part of the plant’s adaptive strategy to enhance barrier properties. These enzymes catalyse the hydroxylation of fatty acids involved in cutin and suberin biosynthesis. This process reinforces the cuticle and suberized tissues, reducing water loss and limiting ion intrusion under salt stress. The cuticle serves a dual role: acting as a physical barrier against biotic and abiotic stresses and containing bioactive compounds, such as phenolics, with antimicrobial activity. Chakraborty et al. [110] confirmed that CYPs are essential not only for cuticle formation but also for detoxifying xenobiotics in plants, insects, and other organisms, as well as in the biosynthesis of secondary metabolites, antioxidants, and phytohormones in higher plants. Given that plant growth and development are constantly challenged by biotic and abiotic stresses, the contribution of *CYP86* genes is particularly significant. Certain *CYP86* genes, along with their downstream targets, enhance stress tolerance through two main mechanisms: (a) promoting the synthesis of protective lipids and waxes—including cutin, suberin, and wax esters—which strengthen the plant’s outer layers and contribute to cuticle formation, and (b) regulating the production of compatible solutes that mitigate the adverse effects of salt stress. These solutes include osmolytes such as proline, glycine betaine, and sugars like trehalose and raffinose, as well as antioxidants such as ascorbic acid (vitamin C), glutathione, carotenoids, flavonoids, and phenolic compounds.

(C) Long-chain acyl-CoA synthetases (LACS) catalyse the activation of long-chain fatty acids into acyl-Co As, providing crucial precursors for various lipid metabolic pathways, including but not limited to cutin biosynthesis. During cutin formation, these acyl-Co As, are integrated into monomers, which are then polymerised to form the cuticle. The cuticle serves as a protective barrier that minimises water loss and limits pathogen invasion. The following equation summarises the reaction catalysed by LACS enzymes.

$$\mathrm{Long}\text{-}\mathrm{chain}\;\mathrm{fatty}\;\mathrm{acid}+\mathrm{CoA}+\mathrm{ATP}\overset{\mathrm{LACS}}{\to }\mathrm{Long}\text{-}\mathrm{chain}\;\mathrm{acyl}\text{-}\mathrm{CoA}+\mathrm{AMP}+\mathrm{PPi}$$

$$R\text{-}\mathrm{COOH}+\mathrm{CoA}\text{-}\mathrm{SH}+\mathrm{ATP}\overset{\mathrm{LACS}}{\to }\mathrm{LACSR}\text{-}\mathrm{CO}\text{-}S\;\mathrm{CoA}+\mathrm{AMP}+\mathrm{PPi}$$

R-COOH: Long-chain fatty acid (e.g., C16–C20), CoA-SH: Coenzyme A, R-CO-SCoA: The activated long-chain acyl-CoA, and ATP is hydrolysed to AMP + Pyrophosphate (PP_i_).

Environmental stresses markedly affect plant survival by altering the activity of LACS in native plants, such as halophytes and xerophytes [111]. These enzymes play a critical role in lipid biosynthesis, including the synthesis of cuticular wax, which serves as a protective barrier against abiotic and biotic stresses [112]. Changes in LACS activity can modify the structure and permeability of the cuticle, thereby influencing plant responses to environmental stresses like drought, salinity, and pathogens [113].

(D) *Bodyguard* (*BDG*) and *Hothead* (*HTH*) are genes found in *Arabidopsis thaliana*, a well-established model organism for molecular genetic studies. These genes encode distinct proteins involved in cutin biosynthesis. *BDG* encodes an α/β-hydrolase fold protein that plays a role in assembling cutin polymers. In contrast, *HTH* encodes a putative oxidoreductase responsible for modifying cutin precursors. Specifically, *HTH* catalyses the oxidation of ω-hydroxy fatty acids into ω-oxo fatty acids, which are key intermediates in the production of cutin monomers. Both proteins may also help limit pathogen spread by maintaining cuticle integrity.

(E) GDSL lipase-like proteins, such as cutin synthases, play a significant role in cutin biosynthesis. These enzymes facilitate both the hydrolysis and polymerisation of cutin monomers, thereby contributing to the formation of the initial cutin polymer network. Despite this involvement, many GDSL lipase-like proteins are not directly responsible for the primary synthesis of cutin. Instead, they are believed to aid in cuticle maturation and structural modification, functions that might become especially crucial during stress conditions.

#### 3.2.3. ABA Biosynthesis and Its Role as a Stress Hormone

ABA (abscisic acid) is a crucial phytohormone with significant developmental and physiological roles [82]. It is primarily recognised as a stress hormone, accumulating in plants under abiotic stress conditions such as drought, salinity, and freezing [114,115]. In desert plants and halophytes, ABA is pivotal in regulating stomatal behaviour [116]. Mechanistically, ABA prompts ion efflux from guard cells, reducing their turgor pressure and promoting stomatal closure [98]. Under water-deficit conditions, ABA accumulation in leaves leads to partial or complete stomatal closure, thereby reducing transpirational water loss. Although this response is vital for conserving water, it simultaneously limits photosynthesis and can influence susceptibility to pathogen ingress during stress [87].

ABA biosynthesis under stress relies on two crucial metabolites: farnesyl pyrophosphate (FPP) and violaxanthin (Vx). FPP serves as a central precursor for various isoprenoids, including carotenoids, sterols, and ubiquinones. In contrast, Vx plays a role in the xanthophyll cycle, protecting Photosystem II by dissipating excess light energy and reducing photoinhibition [117]. Since ABA formation utilises the terpenoid pathway, it competes with other hormone biosynthetic routes, such as those for cytokinins, brassinosteroids, gibberellins, and strigolactones. All these pathways ultimately originate from acetyl-CoA, a vital metabolite produced via glycolysis, the TCA cycle, and interconnected metabolic fluxes. Consequently, stress adaptation in plants involves a significant diversion of energy and metabolic intermediates, necessitating a balance between survival and growth [118].

ABA biosynthesis shares intermediates with other plant hormones, particularly within the terpenoid pathway, while its catabolism primarily involves oxidation or conjugation reactions. Four main enzyme groups catalyse critical steps in ABA biosynthesis, with their activities significantly influenced by environmental stresses such as drought and salinity. These enzymes include zeaxanthin epoxidase (ZEP) and 9-cis-epoxycarotenoid dioxygenase (NCED), located in plastids, as well as short-chain dehydrogenase/reductase (SDR) and abscisic aldehyde oxidase (AAO), which operate in the cytosol [119]. The stress-induced activation of these enzymes results in increased ABA accumulation, allowing sessile plants to adjust their physiology and improve survival under adverse conditions.

A primary example of this is stomatal regulation: ABA-induced ion efflux from guard cells decreases turgor pressure, leading to stomatal closure. This response reduces transpirational water loss but also limits gas exchange, photosynthesis, and transpiration. Although these trade-offs may increase susceptibility to certain biotic stresses, the restricted stomatal aperture can also act as a barrier to pathogen entry. Notably, enhanced ABA synthesis under stress significantly impacts the physiology and biochemistry of native plants, including halophytes and xerophytes [120,121].

Beyond the canonical biosynthetic pathway, other enzyme families may intersect with ABA-mediated stress responses. For instance, members of the CYP77 family, although not directly involved in ABA biosynthesis, contribute to processes such as cuticle and cell wall modifications that may indirectly affect ABA-related signaling. Under certain abiotic stress conditions, both the core ABA biosynthetic enzymes and, in some cases, CYP77 family members show elevated expression levels. While ABA’s developmental and adaptive functions are well established, the precise role of CYP77 enzymes in ABA-mediated processes remains unclear. Further studies are necessary to elucidate the relationships between the CYP77 family and the canonical ABA biosynthetic machinery [120,122].

#### 3.2.4. The Chemical Constituents of Qatari Native Plants

Several native Qatari plants demonstrate antimicrobial properties that enable them to resist pathogens. Additionally, some of these plants have been traditionally used to treat various ailments, indicating their potential as candidates for pathogen resistance. Table 3 lists some of these plants and their roles in pathogen resistance. However, other species may also possess these properties and warrant further investigation.

Moreover, when plants face pathogen attacks, they produce various chemical compounds—including phytoalexins, callose, and ROS—as part of their defence mechanisms. These compounds depend on signalling molecules such as salicylic acid, jasmonic acid, and ethylene, which coordinate the overall defence response [30,143]. Many of these signalling molecules are phytohormones essential for plant defence and stress adaptation [144]. In native plants, the cuticle contains additional chemical defences like alkaloids, tannins, and other secondary metabolites, which are toxic to pathogens and bolster the plant’s overall immunity.

Considerable attention has been directed toward the internal chemical composition of native plants, which underpins their diverse stress-adaptive strategies. Rizk [37] reported on 301 plant species from 207 genera and 55 families, highlighting the extensive array of chemical constituents crucial to plant physiology and biochemistry in arid and semi-arid conditions. These adaptive strategies often depend on secondary metabolites that play roles in defence, stress signalling, and the accumulation of protective solutes. Notable metabolites include phenolics, such as flavonoids and tannins, which possess antimicrobial and antioxidant properties; alkaloids, primarily associated with anti-herbivory defence; and terpenoids, essential for protection and signalling. Additional metabolite classes comprise steroids, glycosides, coumarins, organic acids, quinones, iridoids, glucosinolates, and cyanogenic glycosides [145].

Several studies have provided detailed insights into these constituents and their roles across different plant groups. For example, Rizk and Al-Nowaihi [80] reported on the phytochemistry of 117 horticultural plant species, while Rizk and El-Ghazaly [38] explored the uses of 184 medicinal and poisonous species. Furthermore, Al-Easa et al. [15] presented comprehensive information on the nutritional values of 162 range plant species. Distinguishing sharply between these groups can be challenging, as some plants serve multiple purposes. For instance, certain range species are edible for livestock but also possess medicinal or toxic properties, posing risks if consumed in excess. *Blepharis* spp., comprising approximately 129 species, is a valuable source of medicinal compounds. Extracts from these plants have demonstrated significant antibacterial, antifungal, anti-ulcer, and cytotoxic activities [146,147], highlighting their therapeutic potential. Interestingly, this plant is grazed by camels but not by sheep or goats [15], a pattern consistently noted in various studies and monographs.

The biosynthesis of these compounds in native plants inhabiting harsh environments likely requires substantial energy and involves numerous intermediate metabolites, highlighting their ecological significance. Furthermore, a wide range of osmolytes, such as proline, glycine betaine, and their derivatives, play critical protective roles under stress conditions. These roles include regulating osmotic balance, maintaining turgor and hydration, stabilising enzymes, and cellular structures, acting as nitrogen and energy reservoirs, and preserving chloroplast integrity to sustain photosynthesis, thereby enhancing tolerance to extreme environmental stress [3,48,59,148,149,150,151]. However, the synthesis of such compatible solutes demands considerable energy and metabolic resources, which could otherwise support growth and tissue development, often resulting in reduced productivity under prolonged stress.

Abiotic stresses, including drought, salinity, and extreme temperatures, can drastically alter chemical composition and disrupt metabolic processes. Maintaining stress-induced pathways requires substantial energy, depletes reserves, and may compromise defence mechanisms, increasing susceptibility to bacterial and fungal pathogens. These physiological adjustments may also diminish the palatability and nutritional value of plants as forage, impacting both ecological interactions and economic utility [152]. Additionally, abiotic stresses reshape metabolic profiles and phytohormone signalling, which can either compromise immunity—thus heightening vulnerability to pathogens—or, in some situations, prime defence responses that enhance resistance [153,154]. Further comprehensive studies are needed to elucidate how stress-induced changes in internal chemical constituents, including the accumulation of compatible solutes, stress hormones, and secondary metabolites, affect plant adaptation and resilience.

## 4. Concluding Remarks

Native plants of Qatar, particularly halophytes and xerophytes, exemplify remarkable survival strategies that enable them to persist in extremely saline, arid, and nutrient-poor environments. These species invest metabolic energy in developing protective structures and biochemical defenses—such as thick cuticles, lignified tissues, compatible solutes, and diverse secondary metabolites—that safeguard them against osmotic stress, ion toxicity, dehydration, and pathogens. Their adaptations embody evolutionary resilience and ecological stability rather than high productivity, allowing survival where few species can thrive. Similarly, just as these plants endure and adapt under harsh conditions, a nation or state cannot sustain growth and success if it remains under constant threat; stability and resilience are essential foundations for progress.

Beyond their ecological importance, these native plants hold significant practical value. Their stress-tolerance genes and metabolic pathways represent valuable resources for crop breeding and biotechnology, offering tools to enhance salinity, drought, and heat tolerance in economically important crops. The integration of such traits through molecular breeding, genomic selection, or gene editing could yield cultivars capable of maintaining productivity under climate-induced stress. Moreover, the bioactive compounds identified in native flora provide opportunities for biotechnological innovation, including the development of natural antimicrobials, antioxidants, and biofertilizers.

Ecologically, many native species contribute directly to desert restoration and soil stabilization, mitigating erosion, improving soil structure, and fostering microhabitat formation. Their ability to grow in saline or degraded soils makes them ideal candidates for rehabilitation of arid lands and sustainable landscaping projects.

The central challenge ahead lies in balancing stress tolerance and productivity. While protective mechanisms often reduce growth, strategic application of biotechnology and breeding can optimize both traits. Continued research on Qatar’s native flora—integrating ecology, genomics, and metabolomics—will be essential for unlocking their full potential. These plants stand as invaluable genetic, biochemical, and ecological resources, forming a foundation for innovative approaches to climate-resilient agriculture, desert ecosystem restoration, and sustainable development in arid regions.

## Figures and Tables

**Table 2:** Desert plants versus mesophyte plants (non-stressed) in terms of some structural and chemical features related to their normal habitats in the Arabian Gulf region.

Features	Desert Plants	Mesophytes (Non-Stressed Plants)	Observations
Thickness of cuticle	Thick and waxy	Thin to moderate	Increase in the lipid content of the cuticle, wax biosynthesis by *CER1* * [92]
Cutin composition	Highly polymerised; includes esters of fatty acids, aliphatic polymers: cutan, stress-adapted	Primarily polyester of hydroxy and/or epoxy fatty acids, less complex and thinner	Less cutin in mesophytes unless under stress [93]
Wax content	High; contains long-chain hydrocarbons	Moderate; composed of simpler compounds	Little information, needs further investigation, more wax per surface area, which reduces non-stomatal water loss under stress conditions [94]
Antimicrobials in the cuticle	Abundant phenolics and terpenoids	Few phenolics and terpenoids (produced mainly during pathogen attack)	Secondary metabolites such as phenolics (such as flavonoids, tannins), and the presence of terpenoids, as protective and signaling roles [95]
Salt crystals in cuticle **	Present	Absent	Salt crystals might prevent microbial attack, unless adapted [96]
Phenolics	High concentration	Low to moderate level	Play roles as antimicrobials and antioxidants [97]
Stress hormones (Abscisic acid; ABA)	Elevated	Normal physiological levels	The presence of ABA to regulate stomatal movements [98]
Osmo-protectants	Present such as proline and glycine-betaine, etc.	Almost absent	The presence of compatible solutes to prevent water loss [99]
Reactive oxygen species (ROS)	Constitutively Active	Induced only under stress	The scavenging systems include ascorbate and glutathione [100]

* *CER1*: Agen found in many plants that encodes an enzyme critical for the biosynthesis of cuticular waxes, particularly alkanes, which are major components of the plant cuticle, ** Among desert plants. only halophytes have salt crystals.

**Table 3:** Selected native plant species from the flora of Qatar [9,10] exhibiting resistance to pathogens.

Species	General Characteristics	Specific Features	References
*Artemisia herba-alba*, Syn. *Artemisia inculta* *	Medicinal plant	Source of active molecules, extracts may be used to treat breast cancer, antibacterial, and possibly for other uses	[123,124]
*Echium horridium* * 67 species	Medicinal plants might contain fatty acids such as palmitic acid	Extracts show antioxidant, analgesic, anxiolytic, anti-inflammatory, antibacterial, and antiviral effects	[125,126,127]
*Leptadenia pyrotechnica* **	Medicinal plant	Produces bioactive compounds with pharmaceutical activities, exhibits antimicrobial properties, extracts can resist certain bacteria species like S. aureus, *E*. *coli*, and B. subtilis, and some fungi species such as *A*. *flavus*, and *F*. *moniliforme*	[128,129]
*Leucas urticifolia* **	Medicinal plants contain phytochemicals, such as lignans, flavonoids, coumarins, steroids, terpenes, fatty acids, and aliphatic long-chain compounds	The presence of phytochemicals with antimicrobial properties, these constituents play roles in economic, social, cultural, and ecological aspects	[130,131,132,133]
*Limonium axillare* **	A huge number of bacterial isolates were obtained from leaves; many secondary metabolites were found in plant tissues that can play roles in biocontrol of microorganisms and contribute to sustainable agriculture	Antifungal activity, hosts fungal endophytes such as *Aspergillus* and *Cladosporium*, a huge number of bacterial isolates were obtained from leaves, while root and bark are sources of antidiabetic compounds	[134,135,136]
*Lycium shawii*, Syn. *Lycium arabicum* **	The most common shrub in Qatar responds phenotypically to water availability, from dried twiggy bare spiny bushes to green leafy plants, medicinal plants, the presence of alkaloids and sterols and terpenes, amino acids, fatty acids, and minerals	It exhibits a wide range of pharmacological properties, including antimicrobial, antioxidant, anti-diabetic, anti-inflammatory, anti-cancer, antitrypanosomal, hepatoprotective, antiplasmodial, and cytotoxic activities, making it a potential candidate for treating malaria through its therapeutic compounds	[137,138]
*Rhanterium epapposum* *	Medicinal plants and extracts show significant activity against bacteria and fungi, and are used to cure skin infections	Extracts show antimicrobial properties and antileishmanial activity	[139,140]
*Ziziphus nummulariais* *	Medicinal plants used in traditional folk medicine, rich in phytochemical constituents with pharmacological properties. These components include alkaloids, flavonoids, saponins, glycosides, tannins, and phenolic compounds	Extracts of this plant show a great deal of antibacterial and antifungal activities, exhibit, help to resist pathogens and treat various types of diseases, including cancer, diabetes, and cardiovascular diseases	[141,142]

* Exhibit antimicrobial properties that help them resist pathogens. ** Used for various ailments, can be possible candidates for pathogen resistance.

## Data Availability

The original contributions presented in the study are included in the article, further inquiries can be directed to the corresponding author.
